# Ferroptosis Resistance: Redundant Antioxidant Networks Are a Barrier to Cancer Therapy

**DOI:** 10.3390/antiox15070860

**Published:** 2026-07-09

**Authors:** Birandra K. Sinha

**Affiliations:** Laboratory of Mechanistic Toxicology, Division of Translational Toxicology, National Institute of Environmental Health Sciences, National Institute of Health, Research Triangle Park, Durham, NC 27709, USA; sinha1@niehs.nih.gov

**Keywords:** resistance, ferroptosis, antioxidants, *GPX4*, FSP1, DHODH

## Abstract

Ferroptosis is an iron-dependent, lipid peroxidation-driven form of regulated cell death that has emerged as a promising strategy for targeting therapy-resistant cancers. However, both intrinsic and acquired resistance to ferroptosis-inducing agents (FINs) limit their clinical efficacy. Here, we propose an integrated framework in which ferroptosis resistance arises from coordinated redox, metabolic, lipid, iron, and transport adaptations that collectively suppress lipid peroxidation and promote tumor survival. Central to this network is the cysteine–glutathione–GPX4 axis, supported by parallel GPX4-independent systems including FSP1–CoQ10, DHODH–CoQ10, GCH1–BH4, and NQO1–NADPH pathways. These antioxidant systems are reinforced by NRF2-driven transcriptional programs, iron sequestration mechanisms, lipid remodeling that reduces polyunsaturated fatty acid availability, and ATP-binding cassette (ABC) transporters that regulate drug and glutathione flux. Tumor heterogeneity further enhances ferroptosis resistance by generating metabolically distinct cellular subpopulations that differ in their susceptibility to lipid peroxidation. We discuss emerging therapeutic strategies designed to overcome these coordinated defense mechanisms, including simultaneous targeting of GPX4 and FSP1, metabolic reprogramming, iron-directed therapies, and nanoparticle-based delivery systems. Collectively, these observations support a systems-level model in which durable ferroptosis-based cancer therapy will require disruption of multiple interconnected resistance mechanisms rather than inhibition of a single molecular target.

## 1. Introduction

Ferroptosis is a regulated form of necrotic cell death characterized by iron-dependent lipid peroxidation [1,2,3]. It is triggered by the failure of glutathione-dependent antioxidant defenses (specifically GPX4). In recent years, interest in ferroptosis has increased due to its ability to eliminate therapy-resistant cancer cells and enhance response to immunotherapy [4]. Notably, distinct tumor lineages undergoing epithelial-to-mesenchymal transition (EMT) or therapeutic de-differentiation develop a highly specialized metabolic state that paradoxically increases their basal susceptibility to iron-dependent lipid peroxidation [5,6,7]. These observations position ferroptosis as a promising strategy for targeting drug-resistant cancers. Drug resistance remains one of the most significant barriers to effective cancer therapy and is responsible for a large proportion of cancer-related deaths. Tumor cells frequently develop multidrug resistance (MDR) through adaptive mechanisms such as increased drug efflux, metabolic detoxification, or target modification.

Sorafenib (C_21_H_16_ClF_3_N_4_O_3_), a small-molecule multikinase inhibitor primarily used for advanced hepatocellular carcinoma, and sulfasalazine (C_18_H_14_N_4_O_5_S), a 5-aminosalicylate and anti-rheumatic drug, were the first clinically approved drugs reported to induce ferroptosis [8,9]. Furthermore, compounds such as lenvatinib and regorafenib also possess documented capacities to trigger iron-dependent lipid peroxidation by downregulating downstream survival proteins like GPX4 or altering the STAT3 axis. Similarly, alternative anti-inflammatory 5-aminosalicylates like mesalamine modulate iron handling and transcriptionally alter ferroptosis susceptibility. Currently, several clinical trials are investigating sulfasalazine for solid tumors in combination with novel iron-based nanoparticles designed to trigger ferroptosis [10,11]. These studies highlight the growing clinical interest in ferroptosis-based therapeutic strategies.

Beyond its direct cytotoxic effects, ferroptosis also interacts with antitumor immunity. Ferroptosis can eliminate immunosuppressive cells within the tumor microenvironment and enhance immune-mediated tumor clearance [12,13]. However, the effects of ferroptosis on immune cells are context-dependent and can promote or suppress antitumor immunity depending on cell type and timing.

Despite this interest and promise, clinical translation of ferroptosis inducers remains limited by robust resistance mechanisms that allow tumor cells to maintain redox balance and membrane integrity under oxidative stress. Understanding these resistance networks and the identification of new targets are crucial for the development of ferroptosis-based cancer therapies to overcome ferroptosis resistance.

While prior reviews have focused primarily on individual ferroptosis defense pathways, less attention has been given to how these systems function as coordinated and spatially compartmentalized resistance networks within heterogeneous tumors. Here, we propose that ferroptosis resistance is governed by an integrated, multi-layered network consisting of four major functional modules that buffer lipid peroxidation through compartmentalized and compensatory pathways: (A) redox detoxification (GPX4, FSP1, DHODH, NQO1, GCH1), (B) metabolic rewiring (NADPH synthesis, the pentose phosphate pathway, and lipid remodeling), (C) transport regulation (xCT and ABC transporters), and (D) iron/lipid substrate control. Together, these interconnected modules maintain membrane integrity, suppress lipid peroxidation, and enable tumor survival under ferroptotic stress.

A redox network model of ferroptosis resistance centers on balancing iron-dependent lipid peroxidation with robust antioxidant systems, primarily the system-GSH-GPX4 axis. Resistance is maintained by preventing excessive reactive oxygen species (ROS) accumulation and maintaining membrane integrity through GPX4 activity, which reduces lipid peroxides. Several components of this redox network play significant roles in ferroptosis resistance, and these are discussed below.

## 2. Redox Detoxification Networks

### 2.1. System-GSH-GPX4 Axis

**Figure 1:**
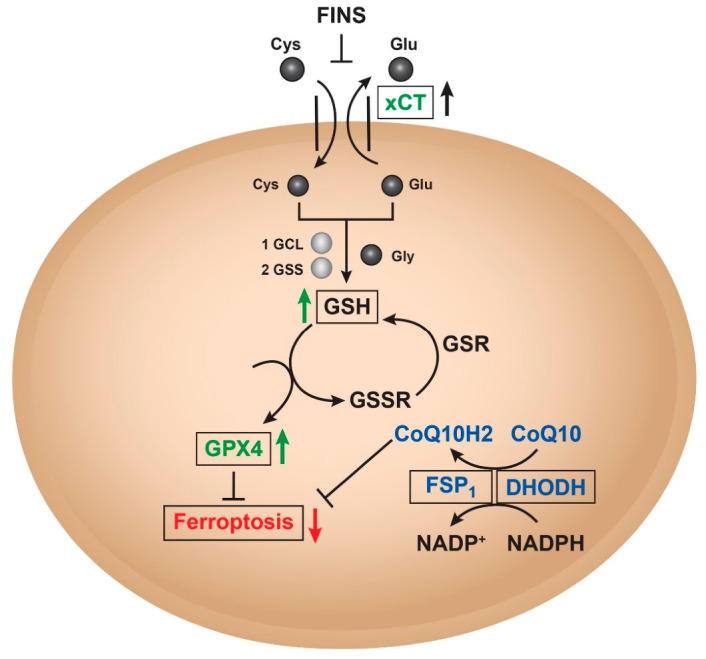
Dual antioxidant systems drive ferroptosis resistance. System xCT imports cystine in exchange for glutamate, supporting glutathione (GSH) synthesis. GSH sustains GPX4 activity to detoxify lipid peroxides and inhibits ferroptosis. In parallel, FSP1 reduces coenzyme Q10 (CoQ10) to CoQ10H_2_ using NADPH, providing an independent lipid antioxidant pathway. Upregulation of both GPX4- and FSP1-dependent systems enhances ferroptosis resistance. Sites of action of FINs are shown in green, and the resistance bypass mechanism is depicted in blue.

The primary defense, importing cystine via glutathione synthesis, which GPX4 uses to reduce lipid hydroperoxides and has been studied extensively [14]. It is a chloride-dependent and sodium-independent antiporter of Cys and Glu, consisting of catalytic subunit xCT/Solute Carrier Family 7 Member 11 (SLC7A11) and regulatory subunit 4F2 (4F2hc)/Solute Carrier Family 3 Member 2 (SLC3A2) connected by disulfide bonds [15,16]. Activation of SLC7A11 expression enables cells to restore redox homeostasis and maintain survival under stressful conditions such as oxidative stress, amino acid starvation, metabolic stress, and genotoxic stress [17]. Thus, System xCT functions as the upstream determinant of GPX4-dependent redox capacity [18,19]. There are many compounds that interfere with System xCT, such as erastin and its analogs, that can lead to cysteine deprivation, glutathione depletion, endoplasmic reticulum stress, and cell death [20,21] (Figure 1). System xCT is an important target for inducing ferroptosis and provides a new direction for the treatment of drug-resistant solid tumors [22].

Extracellular cystine is imported in exchange for intracellular glutamate (Glu) via the cystine/glutamate antiporter system (xCT) for glutathione (GSH) synthesis that supports GPX4 to detoxify lipid peroxides, thereby suppressing ferroptosis. High expression or activity of the GSH-GPX4 axis confers resistance to FINs. Interestingly, several drug-resistant cancer cells have been found to be more sensitive to lipid peroxidation, and inhibitors of the System xCT/GSH/GPX4 axis have been shown to be cytotoxic to these cells [23,24,25]. Cheng et al. found that inhibiting system xCT with erastin enhances the antitumor effect of cisplatin [26].

### 2.2. FSP1-CoQ10H_2_ Pathway

A parallel, glutathione-independent system, Ferroptosis Suppressor Protein 1 (FSP1, also known as AIFM2), acts as a potent oxidoreductase enzyme and protects against ferroptosis by reducing extra-mitochondrial CoQ10 to its active antioxidant form, CoQ10H_2_, using NAD(P)H as a cofactor at the plasma membrane [27]. This reduced CoQ10H2 acts as a lipophilic radical-trapping antioxidant that intercepts phospholipid peroxyl radicals, preventing lethal lipid peroxidation and conferring resistance to ferroptosis [28]. FSP1 uses NADPH to regenerate reduced CoQ10 from its oxidized form, sustaining the antioxidant capacity. Crucially, the FSP1 pathway functions as a robust cell survival backup system capable of shielding cell structures from lethal peroxidation cascades even during total functional collapse or genetic ablation of GPX4. This standalone protection relies on the precise myristoylation-dependent anchoring of FSP1 directly to non-mitochondrial membrane networks and acts as the critical enzyme to reduce ubiquinone to ubiquinol, thereby restoring the reduced pool. FSP1 is considered a critical, druggable target in cancer therapy [29]. Interplay between FSP1 and CoQ10H_2_ in ferroptosis resistance is shown in Figure 1.

### 2.3. Dihydroorotate Dehydrogenase (DHODH)-CoQ10H_2_ Axis

DHODH-CoQ10H2 is a recently discovered antioxidant defense system located in mitochondria that compensates for the loss of GPX4 in detoxifications of mitochondrial lipid peroxides [30]. DHODH is an enzyme involved in pyrimidine synthesis that can reduce CoQ10 to CoQ10H_2_ in the inner mitochondrial membrane. When GPX4 is inactivated, the flow through DHODH is increased, resulting in increased generation of CoQ10H_2_ that neutralizes lipid peroxidation and defends against ferroptosis in the mitochondria [31].

Cells have developed at least four defense systems that collectively form compartmentalized CoQ10-dependent defenses with different subcellular localizations to detoxify lipid peroxides and protect cells from ferroptosis. Cytosolic GPX4 (GPX4cyto) collaborates with FSP1 on the plasma membrane (and other non-mitochondrial membranes), and mitochondrial GPX4 (GPX4 mito) collaborates with DHODH in mitochondria to neutralize lipid peroxides [32]. Notably, mitochondrial GPX4 and DHODH can functionally compensate for one another to suppress mitochondrial lipid peroxidation. In contrast, cytosolic GPX4 and FSP1 lack this capacity, likely because they are absent from the mitochondrial compartment and consequently have an inability to detoxify lipid peroxides within the inner mitochondrial membrane. These observations underscore the critical importance of subcellular compartmentalization in ferroptosis defense. Beyond its canonical metabolic role, DHODH regenerates CoQ10H_2_ to suppress mitochondrial lipid peroxidation and ferroptosis. Elevated DHODH expression in colorectal, hepatocellular, breast, renal, and brain cancers correlates with poor prognosis, therapy resistance, and immune evasion [33]. Pharmacological inhibition of DHODH disrupts pyrimidine synthesis and redox defense, sensitizing GPX4-low tumors to ferroptosis. Preclinical studies demonstrate synergy between DHODH inhibitors and chemotherapy, radiotherapy, or immune checkpoint blockade [34,35]. DHODH serves as both a metabolic and redox checkpoint in cancer, linking ferroptosis suppression to proliferation and immune escape. Targeting DHODH offers a promising strategy to dismantle cancer resilience, particularly in combination with ferroptosis inducers and immunotherapies [33]. Its role in ferroptosis inhibition suggests that DHODH inhibitors could have two complementary mechanisms of action against tumors: inhibiting de novo pyrimidine nucleotide biosynthesis and enhancing ferroptosis. However, the close relationship between mitochondrial function and ferroptosis, together with the role of DHODH in the mitochondrial electron transport chain, suggests that metabolic reprogramming associated with the Warburg effect may influence the contribution of DHODH to ferroptosis. [36]. Furthermore, emerging evidence suggests a link between DHODH and the cellular GSH pool, which may contribute to the rational design of ferroptosis-based anticancer therapies [36].

### 2.4. GCH1-BH4 Axis

Kraft et al. [37] identified GTP cyclohydrolase 1 (GCH1)—the rate-limiting enzyme for tetrahydrobiopterin (BH4) biosynthesis—as a potent endogenous suppressor of ferroptosis. Acting upstream of the lipid-trapping antioxidant BH4, the GCH1–BH4 axis suppresses ferroptosis independently of the canonical GPX4 pathway. Mechanistically, BH4 protects cellular membranes by preventing the depletion of phospholipids containing polyunsaturated fatty acids (PUFAs), thereby limiting the propagation of lipid peroxyl radicals. Furthermore, GCH1 upregulation orchestrates profound lipidomic remodeling by promoting the accumulation of anti-ferroptotic ether phospholipids while reducing highly peroxidizable arachidonic acid-containing phospholipid species. Consequently, elevated GCH1 expression acts as a powerful metabolic shield, conferring resistance to ferroptosis-inducing agents (FINs) and highlighting the GCH1–BH4 axis as a promising therapeutic target in drug-resistant malignancies.

### 2.5. NQO1-NADPH Axis

The NQO1-NADPH axis acts as a crucial, non-canonical defense mechanism against ferroptosis by maintaining reduced coenzyme CoQ10H_2_ and mitigating lipid peroxidation [37]. NQO1 acts as an oxidoreductase, reducing CoQ10 to CoQ10H_2_, which serves as an antioxidant to neutralize lipid peroxides in the plasma membrane. NQO1 requires an abundant supply of NADPH, which is essential for maintaining the cell’s reduced state and supporting other antioxidant systems like GPX4 and FSP1. In many cancers, NQO1 is up-regulated (often via NRF2 signaling) to protect cells from ferroptosis, contributing to chemotherapy resistance [38]. While high NQO1 generally promotes resistance, it can exhibit complex, expression-level-dependent effects on mitochondrial function. Pharmacological NADPH-dependent activation of NQO1 leads to inhibition of ferroptosis in certain models. High NQO1 expression confers resistance, particularly in KEAP1-deficient cancers, while inhibiting this axis sensitizes cells to ferroptosis [37]. Conversely, targeting NQO1 with specific inhibitors or leveraging NQO1-mediated ROS generation can induce ferroptosis in resistant cancer cells [39]. This axis operates independently of, but is complementary to, the canonical system xCT−GSH-GPX4 pathway [40,41].

Although both NQO1 and FSP1 contribute to CoQ10-dependent antioxidant defense, their relationship to NRF2 and ferroptosis resistance is fundamentally different (Table 1). NQO1 is a canonical NRF2 target whose expression tightly tracks NRF2 activation and primarily supports general redox homeostasis through cytosolic quinone detoxification [42,43,44].

In contrast, FSP1 functions as a ferroptosis suppressor by reducing CoQ10 to CoQ10H_2_ directly at cellular membranes, where lipid peroxidation occurs. While FSP1 expressions can be enhanced by NRF2 in some contexts, it is not strictly NRF2-dependent and can remain functional even when NRF2-regulated glutathione and detoxification pathways are compromised [45]. As a result, NRF2 activation significantly amplifies antioxidant capacity, whereas FSP1 provides a GPX4-independent, spatially optimized mechanism that leads to the inhibition of lipid peroxidation. This distinction explains why NQO1 induction alone often confers only partial protection, while FSP1 upregulation is more strongly associated with robust resistance to ferroptosis-inducing agents such as erastin.

**Table 1:** The major compartmentalized antioxidant defense nodes that govern ferroptosis resistance in malignant cells. These survival networks operate across distinct subcellular structures—including the cytosol and plasma.

Pathway	Cellular Localization	NRF2 Dependence	Biochemical Consequence	Reference(s)
**GSH-GPX4**	Cytosol and Mitochondria	**Yes** (Indirect/Direct)	Catalyzes reduction of phospholipid hydroperoxides to non-toxic alcohols using GSH.	[46,47]
**FSP1-CoQ_10_**	Plasma/Extramitochondrial Membranes	**Partial** (Context-dependent)	Utilizes NAD(P)H to continuously regenerate reduced CoQ10H_2_, an active lipid radical-trapping antioxidant.	[25,27,45]
**DHODH-CoQ_10_**	Inner Mitochondrial Membrane	**No**	Regenerates mitochondrial CoQ10H_2_ to inhibit lipid peroxidation.	[30,31,33]
**NQO1-NADPH**	Cytosol	**Yes**	Drives multi-substrate quinone detoxification and supports extramitochondrial quinone reduction.	[38,42,43]

FSP1 sits directly at membranes and continuously regenerates CoQ10H_2_ exactly where lipid radicals form. Even though NRF2 can increase FSP1 expression in some cells, FSP1 does not depend on NRF2 to function [45]. Once FSP1 is present in cells, it acts independently of glutathione and GPX4. It is believed, therefore, that ferroptosis resistance persists even when NRF2-driven pathways are weakened—as FSP1 provides a more direct, localized, and GPX4-independent defense against lipid peroxidation. Together, these systems form spatially compartmentalized antioxidant defenses in cytosol (GPX4 and NQO1), membrane (FSP1) and mitochondria (DHODH and GPX4 mito), protecting tumor cells from ferroptosis and are summarized in Table 1.

NRF2 is a master regulator of antioxidant defenses and iron metabolism that provides robust resistance to ferroptosis [48]. NRF2 coordinates redox and iron homeostasis through transcriptional upregulation of genes involved in glutathione synthesis (SLC7A11, GPX4), iron storage (ferritin), and iron export (ferroportin) [46,47,49,50]. NRF2 enhances cysteine uptake and glutathione synthesis by activating the SLC7A11 subunit of the system xCT transporter, ensuring a steady supply of cysteine for GSH synthesis. NRF2 also directly regulates GPX4 expression [47] and integrates antioxidant, iron and lipid metabolic programs [50,51]. It also enhances the activity of enzymes like glutathione-S-transferases (GSTs) [48]. In many cancers, high NRF2 activation drives resistance to therapies by preventing the accumulation of iron and oxidative damage [50,52]. Consequently, targeting the NRF2 pathway can sensitize resistant cancer cells to ferroptosis-based therapies.

## 3. Metabolic Rewiring and Lipid Remodeling

### 3.1. Iron Metabolism in Ferroptosis Resistance

Iron plays a critical role in redox reactions and mediates oxidative stress, which is considered a driving force in evolution. Dysregulation of iron levels, whether elevated or reduced, can result in cellular and tissue damage, leading to various pathological conditions. Iron is a central driver of ferroptosis, and resistance to this form of cell death is achieved by maintaining low levels of cytoplasmic labile iron (Fe^2+^) and promoting its storage or efflux [53].

Excess intracellular Fe^2+^ is stored by ferritin, a protein complex composed of ferritin light chain (FTL) and ferritin heavy chain 1 (FTH1), preventing it from being oxidized to Fe^3+^. NCOA4 (Nuclear receptor coactivator 4), a cargo receptor protein, binds to ferritin, which is then escorted to the autophagosome for lysosomal degradation (ferritinophagy), leading to the release of Fe^2+^ in the cytosol [54]. Thus, a decrease in the expression of NCOA4 and/or an increase in ferritin expression will result in reduced intracellular free Fe^2+^, causing ferroptosis resistance [54]. Additionally, overexpression of mitochondrial ferritin (FTMt), an iron-storage protein in mitochondria, decreases intracellular free Fe^2+,^ leading to the inhibition or resistance to ferroptosis [55].

Ferroptosis resistance results from coordinated regulation of iron metabolism and antioxidant defenses, including reduced TfR1-mediated iron uptake, enhanced ferritin-dependent iron sequestration, and inhibition of lipid peroxidation by the GSH–GPX4 pathway. By limiting the labile iron pool (LIP) and intracellular Fe^2+^ availability, cells reduce the pool of redox-active iron necessary to propagate ferroptotic lipid damage [56]. In some senescent cells, trapping iron within lysosomes prevents it from triggering oxidative damage in the cytoplasm [57,58]. The iron exporter ferroportin (FPN) reduces intracellular iron levels, thereby reducing the sensitivity to ferroptosis inducers. It has been suggested that iron metabolism works closely with antioxidant systems, such as GPX4, which neutralizes lipid peroxides formed in iron-dependent processes [59]. Strategies that reduce iron availability decrease oxidative stress and accumulation of lipid peroxides.

In addition to these cell-autonomous iron regulation pathways, recent studies reveal that tumor cells can actively purge iron stores to evade ferroptotic stress. Beyond traditional ferroportin-mediated export, cancer cells utilize extracellular vesicle trafficking to lower their intracellular LIP. Specifically, CD63-mediated exosomal secretion of ferritin-bound iron has emerged as a distinct mechanism driving ferroptosis resistance, particularly characterized in ovarian cancer cells [60]. By upregulating the tetraspanin CD63, tumor cells drive the assembly and secretion of multivesicular bodies heavily loaded with iron-rich ferritin complexes. This active external dumping effectively starves the intracellular environment of the redox-active free iron required to power Fenton chemistry. Consequently, this vesicle-mediated expulsion couples systemic microenvironmental intercellular communication directly with individual tumor cell survival, serving as a critical mechanism of therapeutic escape.

Resistance to ferroptosis, therefore, is strongly influenced by the regulation of cellular iron homeostasis. Cells can induce resistance by limiting the availability of the LIP via decreased iron uptake, increased iron storage in ferritin and mitochondrial ferritin, reduced ferritinophagy via NCOA4 suppression, enhanced iron export through ferroportin, and sequestration of iron within intracellular compartments such as lysosomes. These mechanisms collectively reduce the levels of redox-active Fe^2+^ for lipid peroxidation. In parallel, antioxidant systems cooperate with iron-regulatory networks to neutralize lipid peroxides and prevent oxidative damage. Therefore, the interplay between iron metabolism and antioxidant defenses represents a key determinant of ferroptosis sensitivity and a critical mechanism underlying ferroptosis resistance.

### 3.2. Lipid Metabolism in Ferroptosis Resistance

Because lipid peroxidation is considered the execution step of ferroptosis, remodeling membrane lipid composition is a dominant resistance mechanism. Lowering polyunsaturated fatty acid (PUFA) content or modifying membrane compositions (e.g., via ACSL4 inhibition) reduces substrate availability for lipid peroxides. In cancer cells, ELOVL5 (Elongation of Very Long Chain Fatty Acids Protein 5) regulates ferroptosis resistance primarily by modulating the synthesis of long-chain polyunsaturated fatty acids (LC-PUFAs), which are substrates for lipid peroxidation [61,62]. In many cancer cells, high ELOVL5 expression promotes sensitivity to ferroptosis by producing arachidonic acid (AA) and adrenic acid (AdA), while its downregulation or silencing leads to resistance [62,63]. In breast cancer cells, knockdown or low expression of ELOVL5 decreases polyunsaturated fatty acids in the cell, preventing the formation of toxic lipid peroxides and thus protecting against ferroptosis [64]. ELOVL5 generally promotes ferroptosis sensitivity through PUFA synthesis, although in certain oncogenic contexts it can contribute to therapy resistance via lipid signaling pathways (e.g., AKT–mTOR activation) [65]. ACSL4 is a key promoter of ferroptosis, while ACSL3 often plays a protective role against it [66,67]. ELOVL5-derived fatty acids are incorporated into membrane phospholipids and lipid droplets via ACSL3 and ACSL4, influencing ferroptosis susceptibility [68]. Studies show that when ELOVL5 is silenced, the levels of arachidonic acid and eicosapentaenoic acid decrease, directly affecting the substrate availability for ACSL4-mediated activation [69]. ELOVL5-derived PUFAs are esterified into phospholipids by ACSL4, while ACSL3 promotes MUFA incorporation into lipid pools, including lipid droplets, collectively shaping ferroptosis susceptibility [70].

While ACSL4 promotes ferroptosis by enabling accumulation of oxidized phospholipids in cellular membranes, SCD1 (Stearoyl-CoA desaturase 1) protects against ferroptosis by converting saturated fatty acids (SFAs) into monounsaturated fatty acids (MUFAs), reducing the accumulation of lipid peroxides [71]. SCD1 is responsible for the biosynthesis of monounsaturated fatty acids for maintaining membrane fluidity, cellular signaling, and gene expression [72,73]. SCD1 catalyzes the D9-cis desaturation of a range of fatty acyl-CoA substrates. The preferred substrates are palmitoyl- and stearoyl-CoA, which are converted into palmitoleoyl- and oleoyl-CoA, respectively. Endogenous synthesis is the key driver in cancer cells of oleate, and is the most abundant monounsaturated fatty acid [74]. SCD1 has been reported to be involved in cancer stem cells in various types of cancer, and therefore, SCD1 has been suggested as a novel therapeutic target for cancer treatment [75]. Various natural products can inhibit SCD1 expression/activity, thereby suppressing cancer cell survival and self-renewal activity [76]. Mechanistically, upregulated expression of the monounsaturated fatty acid (MUFA) stearoyl-CoA desaturase alters the ratio of MUFA-PLs to PUFA-PLs, leading to ferroptosis resistance.

Overexpression of LPCAT1 (lysophosphatidylcholine acyltransferase 1) is associated with various cancers, including colorectal, prostate, and renal cancers, where it promotes tumor growth, migration, and invasion [77]. LPCAT1 catalyzes the conversion of lysophosphatidylcholine (LPC) to phosphatidylcholine (PC) by adding a palmitoyl group. Interestingly, LPCAT1 has been shown to be involved in ferroptosis resistance by enhancing membrane phospholipid saturation through the Lands cycle [78]. Furthermore, inhibition of LPCAT1 in the presence of ferroptosis inducers synergistically enhanced ferroptosis and suppressed tumor growth in preclinical models, suggesting that LPCAT1 could be a target for ferroptosis-inducing therapies in cancer treatment [78]. Furthermore, it was shown that silencing of LPCAT1 decreased saturated fatty acid-containing phospholipid levels, resulting in sensitization of the ferroptosis-resistant cancer cells to ferroptosis. In contrast, overexpression of LPCAT1 caused an increase in ferroptosis resistance, due to incorporation of SFAs (exogenous or endogenous) but not MUFAs [78,79].

Thus, the resistance to ferroptosis is governed by lipidomes of cellular membranes, and differences in membrane PUFA content or regulation of cellular PUFA content determine sensitivity or resistance to ferroptosis. Cells can synthesize MUFAs (e.g., oleic acid) and incorporate them into membrane phospholipids, diluting the concentration of peroxidizable lipids [80]. Additionally, decreasing cellular PUFA decreases susceptibility to ferroptosis, causing cellular resistance to lipid oxidation. Xin et al. have suggested that specific changes, such as the synthesis of shorter, more saturated ether lipids, isolate cells from reactive oxygen species and restrict lipid elongation [81]. Cells have been shown to utilize metabolic proteins, e.g., DGAT1 (diacylglycerol-acyltransferase 1), to transport potentially harmful, unbound fatty acids away from the membrane and sequester them safely into lipid droplets, reducing cellular lipid peroxidation [82].

Collectively, these adaptive alterations in lipid metabolism and membrane composition highlight the central role of the cellular lipidome in determining ferroptosis sensitivity. As such, lipid remodeling not only serves as a critical mechanism of ferroptosis resistance but also represents an important link between cellular metabolic state and susceptibility to oxidative cell death.

Roles of lipid pathways and metabolism in ferroptosis resistance are summarized in Table 2.

## 4. Miscellaneous

### 4.1. ABC Transporter-Mediated Resistance

ATP-binding cassette (ABC) transporters utilize ATP hydrolysis to transport various compounds across cell membranes against concentration gradients [83]. Drug-resistant cancer cells usually express these transporters. Furthermore, heterogeneous tumors, e.g., cancer stem cells, also express these transporters, making them difficult to eradicate during chemotherapy. ABC transporters often contribute to ferroptosis resistance by controlling substrate availability and drug exposure. Frye et al. (2023) have reported that P-glycoprotein (P-gp, ABCB1) confers resistance to several erastin-derivatives, offering protection from ferroptotic cell death [84]. Erastin has been reported to inhibit functions of ABC transporters, resulting in increased intracellular concentrations of drug and enhancing drug-induced ferroptosis [24,85].

High ABCC2 (MRP2) plays a significant role in amino acid metabolism and ferroptosis by mediating GSH efflux in gastric cancer, resulting in alterations in redox status, and increasing the cell’s susceptibility to ferroptosis [86]. De Souza et al. have reported that NRF2 positively correlates with ABCC1 expression in tumor tissues of glioma patients, which can be associated with tumor aggressiveness, drug resistance, and poor overall survival [87]. High NRF2 levels promote chemotherapy resistance by upregulating ABCC1, which then acts as an efflux pump to remove drugs from cancer cells. Altogether, studies indicate that high levels of NRF2 result in collateral sensitivity to glioblastoma via the expression of its pro-ferroptotic target ABCC1, leading to GSH depletion when the system xCT is blocked by erastin, weakening the cell’s antioxidant defenses. Thus, ferroptosis induction could be an important therapeutic strategy to reverse drug resistance in gliomas with high NRF2 and ABCC1 expressions.

ABCC5 has been identified as a critical regulator and a promising therapeutic target of acquired sorafenib resistance in human hepatocellular carcinoma cells [88]. The expression of ABCC5 was dramatically induced in sorafenib-resistant HCC cells and was associated with poor clinical prognoses. The downregulation of ABCC5 expression significantly reduced the resistance of sorafenib to HCC cells. ABCC5 increased intracellular glutathione and attenuated lipid peroxidation accumulation by stabilizing SLC7A11 protein, inhibiting ferroptosis. Inhibition of ABCC5 enhanced the anticancer activity of sorafenib in vitro and in vivo, indicating that ABCC5 played a significant role in ferroptosis resistance.

### 4.2. Tumor Heterogeneity in Ferroptosis Resistance

Tumor heterogeneity (TH) exists at both the cellular and tissue levels and plays a critical role in determining cell survival, cell death, and resistance to anticancer therapies [89]. The complex and evolving nature of tumors contributes significantly to resistance against conventional cancer treatments [90]. The emergence of drug-resistant cancer cell populations provides selective advantages, including enhanced proliferation, survival, immune evasion, and cellular plasticity. Recent studies have shown that dysregulated lipid metabolism and iron homeostasis are key contributors to ferroptosis resistance in cancer cells [91].

Tumor heterogeneity is a major driver of ferroptosis resistance because it generates subpopulations of cells with intrinsic or acquired mechanisms that protect against lipid peroxidation, enabling them to evade treatment-induced cell death. These resistant populations often exhibit distinct metabolic, genetic, or differentiation states that promote survival and contribute to tumor recurrence [92]. Key mechanisms through which heterogeneity promotes ferroptosis resistance are discussed below.

### 4.3. Distinct Cellular Differentiation States and Phenotypes

Tumor subpopulations with different differentiation states can exhibit markedly different sensitivities to ferroptosis. In breast cancer, single-cell RNA sequencing (scRNA-seq) studies have demonstrated that luminal-differentiated cells are significantly more resistant to ferroptosis than basal-like, highly proliferative cells [93]. This resistance is largely driven by the transcription factor GATA3, a key regulator of luminal differentiation. GATA3 promotes ferroptosis resistance by reducing the expression of human integrin α1β1, which subsequently decreases the expression of ACSL4 (acyl-CoA synthetase long-chain family member 4), resulting in membrane lipid compositions that are less susceptible to peroxidation [94].

Furthermore, elevated GATA3 expression has been shown to suppress the ferroptosis-related gene CYB5R2 and reduce intracellular iron levels, thereby increasing resistance to doxorubicin-mediated ferroptosis in breast cancer cells [95]. Because high GATA3 expression is indicative of a strongly differentiated luminal phenotype, it is increasingly used as a prognostic biomarker. In combination with genomic profiling, GATA3 status can help guide treatment de-escalation strategies by identifying patients who may benefit from hormone therapy alone rather than toxic chemotherapy. Conversely, Querzoli et al. suggested that GATA3 immunohistochemistry (IHC) may identify breast cancer patients within low-risk categories who have poorer clinical outcomes and could benefit from additional tailored therapies [96].

### 4.4. Metabolic Heterogeneity

Metabolic heterogeneity enables certain tumor cell populations to maintain redox homeostasis and thereby resist ferroptotic cell death [97]. For example, specific melanoma subpopulations can reprogram their metabolism to utilize lactate as an energy source, increasing intracellular NADH and NADPH levels and strengthening antioxidant defenses [98]. Similarly, increased pentose phosphate pathway activity in certain tumor sub-clusters generates the NADPH required to support major ferroptosis defense systems, including the GPX4/GSH pathway and the FSP1/CoQ10 system [98].

As a result, ferroptosis-inducing therapies may selectively eliminate sensitive cells while allowing pre-existing resistant populations to survive and expand, ultimately leading to disease recurrence. These adaptive responses highlight the importance of metabolic diversity as a mechanism of ferroptosis resistance.

Importantly, metabolic heterogeneity does not arise in isolation but is strongly influenced by the tumor microenvironment. Spatial variations in oxygen availability, nutrient supply, and metabolite accumulation create distinct ecological niches within tumors, driving the emergence of subpopulations with unique metabolic adaptations and ferroptosis sensitivities. Consequently, the tumor microenvironment serves as a major determinant of ferroptosis resistance through its effects on both cellular metabolism and phenotypic plasticity.

### 4.5. Tumor Microenvironment in Ferroptosis Resistance

The tumor microenvironment (TME) is a major contributor to tumor heterogeneity, influencing cellular differentiation, epithelial–mesenchymal plasticity, and metabolic reprogramming. The TME consists of diverse non-malignant cell types, including macrophages, T cells, fibroblasts, and other stromal cells, which interact dynamically with cancer cells to create a highly heterogeneous cellular landscape.

Structural abnormalities within tumors often lead to regions of restricted blood flow and oxygen deprivation, resulting in hypoxia. The role of hypoxia in ferroptosis resistance is highly context-dependent and can either promote resistance or sensitize cells to ferroptosis, depending on the tissue type, duration of oxygen deprivation, and underlying oncogenic signaling pathways [99,100]. Reduced oxygen availability may limit the formation of lipid peroxyl radicals, thereby decreasing susceptibility to ferroptosis. In many solid tumors, including pancreatic cancer, hypoxia promotes ferroptosis resistance through stabilization of HIF-1α and HIF-2α, which activate antioxidant pathways such as NRF2/HO-1 and increase cysteine availability for glutathione synthesis, thereby sustaining GPX4 activity [100,101].

Hypoxia also regulates iron metabolism. In certain cellular contexts, it inhibits ferritinophagy, reducing intracellular free iron levels and consequently suppressing ferroptosis [54]. Additionally, hypoxia-induced alterations in lipid metabolism have been shown to confer ferroptosis resistance in prostate cancer cells [102].

Beyond hypoxia, tumor heterogeneity generates regions characterized by nutrient deprivation and extracellular acidification. This acidic microenvironment arises primarily from enhanced glycolytic activity and the accumulation of lactic acid. Tumor acidosis has a dual role, promoting both therapeutic vulnerabilities [103] and adaptive survival mechanisms that facilitate tumor progression and treatment resistance [104]. Consequently, these microenvironmental differences contribute to the emergence of tumor subpopulations with varying sensitivities to ferroptosis and an increased capacity for therapeutic escape.

A deeper understanding of tumor heterogeneity and its role in ferroptosis resistance may enable the development of more effective therapeutic strategies. Combination approaches that pair ferroptosis inducers, such as GPX4 inhibitors, with agents targeting specific resistance mechanisms—including immunotherapies, lactate uptake inhibitors, or modulators of the tumor microenvironment—may be necessary to eliminate both ferroptosis-sensitive and ferroptosis-resistant tumor subpopulations.

**Figure 2:**
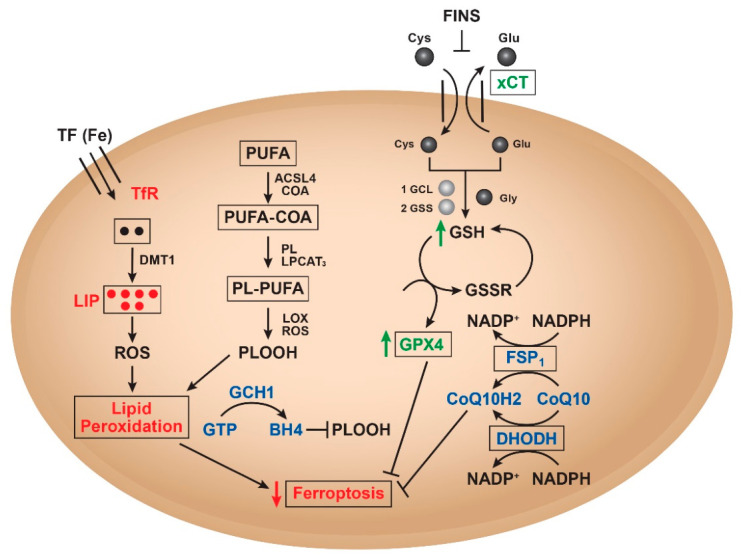
Integrated model of ferroptosis resistance in cancer. Canonical and non-canonical antioxidant pathways suppress lipid peroxidation and ferroptotic cell death. The xCT–GSH–GPX4 axis detoxifies phospholipid hydroperoxides, while FSP1–CoQ10H_2_ and mitochondrial DHODH–CoQ10H_2_ provide GPX4-independent protection. The GCH1–BH4 pathway further limits lipid peroxidation. Iron uptake through TfR and the labile iron pool promotes ROS generation and PUFA-containing phospholipid oxidation. Ferroptosis inhibitors block cystine uptake through xCT. Together, these pathways regulate ferroptosis sensitivity and tumor cell survival. Sites of action of FINs are shown in green, and the resistance bypass mechanism is depicted in blue.

The integrated model of ferroptosis resistance is summarized in Figure 2.

## 5. Future Directions and Therapeutic Considerations

Despite advances, key challenges remain in modeling and detecting ferroptosis resistance in vivo. Current limitations include the lack of validated biomarkers and targeted delivery of FINs and inhibitors without off-target toxicity. It has been suggested that conditional GPX4 knockout mice can be utilized to study how cells adapt or resist ferroptosis through alternative pathways [105]. Also, one can utilize NRF2-overexpressing models that mimic the high resistance observed in some cancers [106,107]. Additionally, tumor or xenograft models overexpressing SLC7A11 for resistance due to cystine-deprivation-induced ferroptosis can be utilized [108]. However, tumors in vivo are more complex and often show more than one pathway for resistance, as found in certain breast cancer cells [25]. It is also possible that in vivo resistance may result from the presence of both ABC transporters and enhanced metabolic detoxification of FINs. These scenarios further complicate the choice of models of ferroptosis resistance. Furthermore, it has recently been reported that both 3D and in vivo tumor models show significant resistance to lipid peroxidation due to extensive lipid remodeling [80], adding additional complications for studying the mechanism of resistance.

With regard to biomarkers, at this time, there are no valid biomarkers that can be utilized as predictive of ferroptosis resistance. However, levels of 4-HNE or MDA, phospholipid hydroperoxides (PLOOH), or reduced glutathione can be measured by mass spectrometers. The Western blots or RT-PCR methods can also be utilized to detect elevated levels of GPX4, FSP1, or SLC7A11 in both in vitro and in vivo. However, these limitations highlight the need for standardized ferroptosis-resistance models and validated biomarkers to enable clinical translation.

Targeted delivery of FINs or inhibitors of FSP1 presents another difficult task, as high concentrations of these compounds are toxic, resulting in serious adverse effects [109]. Doxorubicin (DOX) is an extremely effective drug for the treatment of many human cancers in the clinic [110]. Doxorubicin is a well-known ROS generator and induces lipid peroxidation for both its antitumor activities and cardiotoxicity [111,112]. Liposomal encapsulation of DOX has been shown to decrease its cardiotoxicity and enhance its anti-tumor activity, especially in liposomes prepared from unsaturated phospholipids, which become incorporated and provide an abundance of lipid molecules for lipid peroxidation [113,114]. It is believed that nanoparticle-based delivery systems enhance therapeutic efficacy by simultaneously targeting multiple ferroptosis defense arms while reducing toxicity. Encapsulation of various FINs not only can improve their solubility but may also result in delivering a higher concentration directly to the cellular membrane, resulting in enhanced effects. Similarly, encapsulated inhibitors of FSP1 can also be effectively delivered to tumor sites, increasing their selectivity and simultaneously decreasing off- target toxicity.

In this perspective, I propose that ferroptosis resistance in cancer is governed by an integrated network of redox-regulatory, metabolic, and transport adaptations that suppress lipid peroxidation and sustain tumor cell survival. Collectively, these pathways form a spatially and functionally compartmentalized network that suppresses lipid peroxidation through redundant antioxidant buffering. These antioxidant pathways are further supported by NRF2-mediated transcriptional programs, iron sequestration, and lipid remodeling that reduces polyunsaturated fatty acid availability, and ABC transporters that regulate drug and glutathione flux.

Our recent work further demonstrated that ferroptosis resistance can arise through distinct pathway-specific antioxidant programs. In parental MCF-7 breast cancer cells, resistance to erastin is largely mediated by activation of the FSP1–CoQ10–NADPH pathway together with upregulation of the GSH–GPX4 antioxidant system, resulting in reduced lipid peroxidation and suppression of ferroptotic death. In contrast, the multidrug-resistant MXR derivative relies predominantly on the xCT–GSH–GPX4 axis, making these cells more susceptible to erastin-induced ferroptosis. These findings identify FSP1 as a critical regulator of ferroptosis resistance and suggest that co-targeting GPX4 and FSP1 may represent an effective strategy to sensitize resistant breast cancer cells to ferroptosis-inducing agents.

Importantly, the biological control of ferroptosis should be viewed through the lens of active pathway modulation rather than passive radical scavenging. While direct radical-trapping antioxidants (RTAs) neutralize lipid peroxyl radicals stoichiometrically, an increasingly recognized class of anti-ferroptotic compounds confers protection by enhancing endogenous antioxidant enzymes and regulatory pathways. Central to this response is the Keap1–NRF2 signaling axis. Electrophilic pathway modulators such as oltipraz, sulforaphane, and dimethyl fumarate disrupt the Keap1–NRF2 complex, promoting NRF2 nuclear translocation and the transcriptional upregulation of key ferroptosis defense genes, including *SLC7A11*, *GCLC/GCLM*, and *GPX4*. Complementing this transcriptional regulation, nutritional modulators such as selenium enhance the translation and activity of selenoproteins, particularly GPX4, thereby strengthening cellular defenses against lipid peroxidation. Together, these findings emphasize that ferroptosis resistance is governed by coordinated, context-dependent antioxidant networks rather than isolated defense mechanisms.

Overall, these advances reinforce the concept that ferroptosis resistance is driven by complex, interconnected antioxidant networks that cannot be effectively overcome by targeting a single pathway. Given the redundancy and adaptability of ferroptosis defense mechanisms, combinatorial therapeutic strategies that simultaneously disrupt complementary antioxidant systems are likely to provide the greatest clinical benefit. Continued investigation into the molecular crosstalk governing ferroptosis will be essential for identifying novel therapeutic vulnerabilities and refining precision medicine approaches. Collectively, these findings support a paradigm shift from targeting individual ferroptosis regulators to rationally designing network-directed combination therapies that dismantle the antioxidant defenses sustaining tumor survival. Such strategies hold considerable promise for overcoming therapy resistance and improving outcomes in patients with refractory cancers. The major ferroptosis resistance mechanisms and their corresponding therapeutic targeting strategies are summarized in Table 3, providing a framework for the development of next-generation ferroptosis-based cancer therapies.

## Figures and Tables

**Table 2:** Summary of essential regulatory enzymes in lipid metabolism that dictate whether a cancer cell undergoes ferroptotic death or develops survival adaptation. Enhanced sensitivity is driven by ELOVL5 and ACSL4, which enrich membranes with highly oxidizable PUFAs, while resistance is controlled by SCD1, ACSL3, and LPCAT1 to synthesize monosaturated fatty acids (MUFAs).

Molecular Regulator	Primary Biological Function	Impact on Ferroptosis	Mechanism	References
ELOVL5	Long-chain PUFA elongation	↑ Sensitivity	Expands the pool of oxidizable PUFAs available for membrane incorporation.	[61,62]
ACSL4	PUFA activation and phospholipid incorporation	↑ Sensitivity	Enriches PUFA-containing phospholipids that undergo lipid peroxidation.	[68]
ACSL3	MUFA activation	↓ Sensitivity	Promotes MUFA incorporation, displacing oxidizable PUFAs.	[70]
SCD1	MUFA biosynthesis	↓ Sensitivity	Increases membrane MUFA content and limits lipid peroxidation.	[70,76]
LPCAT1	Phospholipid remodeling (Lands cycle)	↓ Sensitivity	Incorporates saturated fatty acids into membrane phospholipids, increasing membrane saturation and reducing PUFA-dependent lipid peroxidation.	[78,79]

**Table 3:** Major ferroptosis resistance nodes and corresponding therapeutic strategies. Cancer cells evade ferroptosis through multiple adaptive mechanisms that preserve redox homeostasis, restrict lipid peroxidation, regulate iron availability, or reduce intracellular accumulation of ferroptosis-inducing agents. Therapeutic inhibition of these complementary resistance pathways may enhance ferroptosis sensitivity and improve the efficacy of combination anticancer therapies.

Resistance Node	Role in Ferroptosis Resistance	Targeting Strategy	Representative Agents	Clinical Status	References
GPX4	Detoxifies phospholipid hydroperoxides	GPX4 inhibition	RSL3, ML210	Preclinical	[115,116]
FSP1	Regenerates CoQ10H_2_ independently of GPX4	FSP1 inhibition	iFSP1, FSEN1	Preclinical	[25,27,117]
DHODH	Mitochondrial antioxidant defense	DHODH inhibition	Brequinar	Clinical drug repurposing	[118]
SLC7A11 (xCT)	Maintains cystine uptake and GSH synthesis	xCT inhibition	Sulfasalazine	Clinical drug repurposing	[119,120]
ABC transporters	Efflux ferroptosis inducers	Transport inhibition	Tariquidar, Cyclosporine, PSC833	Preclinical	[121,122]
Lipid remodeling enzymes	Reduce oxidizable phospholipids	ACSL4 activation, SCD1 inhibition	Experimental	Preclinical	[66,71]
Iron metabolism	Restricts labile iron pool	Iron-based therapies	Artesunate, DHA, Iron nanoparticles	Clinical/Preclinical	[11,123,124]
CD63-mediated exosomes	Export ferritin-bound iron	Exosome inhibition	GW4869, CD63 antibody	Experimental	[125]

## Data Availability

Data are contained within the manuscript and are available upon request.

## Abbreviations

GPX4, Glutathione Peroxidase 4; CoQ10H_2_, Ubiquinol; FSP1, Ferroptosis Suppressor Protein 1; DHODH, Dihydroorotates Dehydrogenase; NQO1, NADPH Quinone Reductase 1; BH4, Tetrahydrobiopterin; GCH, GTP cyclohydrolase 1; NRF2, Nuclear factor erythroid 2-related factor 2; ABC, ATP-binding Cassette, System xCT, cysteine/glutamine antiporter; FIN, ferroptosis inducing agents; ROS, Reactive oxygen species; L-ROS, lipid-derived reactive oxygen species; GATA3, GATA binding protein 3; ACSL3, Acetyl-CoA Synthetase Long Chain family member 3; ACSL4, Acetyl-CoA Synthetase Long Chain family member 4; SLC7A11, Solute Carrier Family 7 Member 11; LIP, Labile iron pool; PUFA, polyunsaturated fatty acid; ELOVL5, elongation of very long chain fatty acids 5; SCD1, Stearoyl-CoA-desaturase; LPCAT1, lysophosphatidylcholine acyl-transferase; DOX, doxorubicin.
